# Multicolor Histochemical Staining for Identification of Mineralized and Non-Mineralized Musculoskeletal Tissue: Immunohistochemical and Radiological Validation in Decalcified Bone Samples

**DOI:** 10.3390/bioengineering9100488

**Published:** 2022-09-21

**Authors:** Yu Sun, Heike Helmholz, Regine Willumeit-Römer

**Affiliations:** 1 Institute of Metallic Biomaterials, Helmholtz-Zentrum Hereon, 21502 Geesthacht, Germany; 2 Department of Orthopaedics, First Hospital of China Medical University, Shenyang 110001, China

**Keywords:** musculoskeletal tissue, fracture callus, cartilage, muscle, histochemistry, immunohistochemistry, radiology

## Abstract

Histochemical staining of paraffin-embedded decalcified bone samples is commonly used in preclinical research of musculoskeletal diseases, enabling the visualization of multiple tissue components by the application of chromogens. The purpose of this study was to introduce a novel multicolor staining protocol involving optimized chemical reagents and procedure, allowing the identification of high-mineralized bone, low-mineralized fracture callus, cartilage and skeletal muscle fibers simultaneously. Fractured femur and healthy tail vertebra samples from adult male Sprague–Dawley rats were decalcified with EDTA and formic acid, respectively, followed by paraffin embedding, tissue sectioning and multicolor staining. Conventional Movat’s pentachrome and safranin O / fast green staining were conducted in parallel for comparison. Immunohistochemical staining of collagen type-X and micro-CT analysis were included to further validate the efficacy of the staining method. The multicolor staining allowed visualization of major musculoskeletal tissue components in both types of decalcified samples, providing quality outcomes with fewer chemical reagents and simplified procedures. Immunohistochemical staining demonstrated its capacity for identification of the endochondral ossification process during fracture healing. Micro-CT imaging validated the staining outcome for high-mineralized skeletal tissue. The application of the multicolor staining may facilitate future preclinical research involving decalcified paraffin-embedded samples.

## 1. Introduction

Non-invasive imaging techniques such as plain film, micro-CT, ultrasonography and magnetic resonance imaging have been successfully introduced in preclinical studies, enabling longitudinal in vivo monitoring of disease progression and treatment effects in musculoskeletal disorders [1,2]. However, traditional ex vivo histo-pathological methods based on microscopy remain essential for translational research, allowing high-resolution rendering of tissue and cellular details, as well as quantitative assays at the gene and protein levels [3,4,5]. For bone samples in histological studies, although non-decalcified sections can be obtained with hard tissue or cryo-sectioning, decalcification and paraffin embedding are still commonly used for sample processing [6]. Once decalcified, paraffin-embedded samples request relatively simple and similar treatment procedures as non-musculoskeletal organs, without significant tissue loss compared with hard tissue cutting and grinding. High-quality continuous ribbons of connected sections can be obtained without using heavy-duty microtome or carbide blade, and long-term sample storage is possible, allowing immunohistochemistry, immunofluorescence, in situ hybridization and proteomics analysis [3,4,5,7,8].

Histochemical staining is the commonly used analytical method for paraffin-embedded samples. In addition to conventional hematoxylin and eosin (H&E) staining, there are specific methods for bone samples such as Movat’s pentachrome and safranin O / fast green staining, supporting qualitative assessment of bone and cartilage, and quantitative analysis including the calculation of area percentage [9,10]. Movat’s pentachrome is a classic protocol providing visualization of bone, cartilage and other soft tissues in different colors. However, it involves a long list of chemical reagents, and a single staining session could take more than 4 hours, which also requires specialized training and expertise to achieve reproducible results [11]. In comparison, safranin O / fast green staining requires fewer chromogens and is less time-consuming, being widely used in studies of traumatic or inflammatory bone and joint disorders. However, for samples after long-term decalcification with ethylenediaminetetraacetic acid (EDTA), the staining may not be as effective as in samples decalcified with other acids such as hydrochloric acid, nitric acid and formic acid [12,13]. There are other protocols for decalcified samples, for example, Goldner’s trichrome, toluidine blue and picrosirius red staining. However, these methods have limitations in staining outcomes for tissue differentiation and quantification, and some also involve multiple chromogens and complicated processing steps [6,9,14].

The purpose of this study was to introduce a novel multicolor histochemical staining protocol for bone samples, involving only two commercially available chromogens and few steps to obtain quality results for the identification of mineralized and non-mineralized tissues. Micro-CT scan and immunohistochemical staining were adopted for validation of the staining outcomes in samples decalcified with EDTA and formic acid. 

## 2. Materials and Methods

### 2.1. Sample Selection

**Figure 1:**
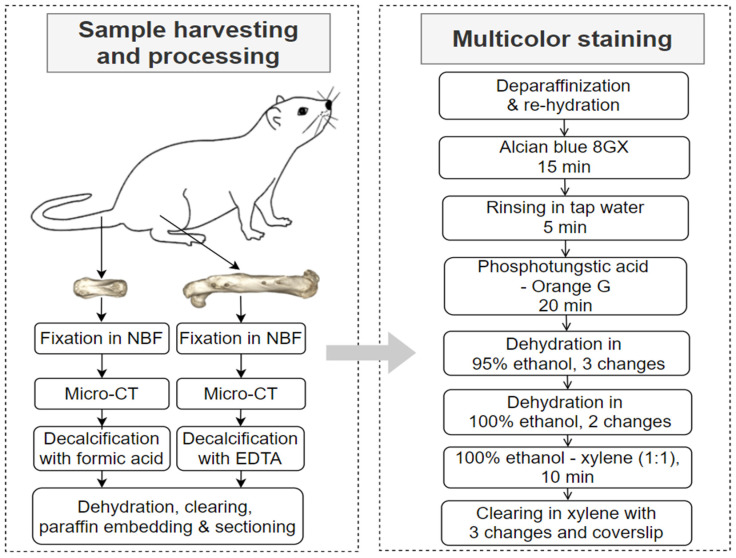
Sample harvesting, processing and the multicolor staining procedure. Note: decalcification solutions: 20% EDTA or 5% formic acid (NBF: neutral-buffered formalin; EDTA: ethylenediaminetetraacetic acid).

The samples used in this study were obtained from preclinical research for establishing a femoral fracture model in male Sprague–Dawley rats. The experimental protocol was applied via the local authority (Ministry for Energy Transition, Agriculture, Environment, Nature and Digitalization, Schleswig-Holstein, Germany, application number: V242-30912/2020), with surgical and related details described in a previous publication [1]. The rats (20 weeks, 515−580 g) were euthanized via overdose anesthesia at post-operative week 12 after femur fractures stabilized by external fixation. The authors selected femur and tail vertebra samples to include the following types of tissue: cortical bone, fracture callus, joint cartilage and skeletal muscle, which were commonly involved in musculoskeletal studies using histochemical staining methods (Figure 1) [9,10].

### 2.2. Sample Processing

The following sample processing procedures were conducted as shown in Figure 1. In brief, the surgical femurs and tail vertebra were harvested and fixed in neutral-buffered formalin for 72 h, followed by ex vivo micro-CT imaging. Then, the femur samples were decalcified with 20% EDTA, and tail vertebra samples were decalcified with 5% formic acid. The endpoint of decalcification was determined by manual checking and X-ray examination (scoutview with micro-CT). Afterwards, the samples underwent dehydration in ethanol gradients, clearing with xylene and paraffin embedding. Paraffin blocks were sectioned using a HistoCore Autocut microtome (Leica Microsystems, Germany) with a thickness of 10 μm.

### 2.3. Histochemical and Immunohistochemical Staining

Consecutive section slides from the femur and vertebra samples underwent a novel multicolor staining (Figure 1) and Movat’s pentachrome staining. Moreover, for femur samples decalcified with EDTA, H&E staining as well as immunohistochemical staining for collagen type-X were conducted as routine procedures for fracture research at the authors’ institute. For vertebra samples decalcified with formic acid, safranin O / fast green staining was conducted as a common procedure for preclinical research of bone and joint diseases such as osteoarthritis. Detailed information of chemical reagents is listed in Table 1, and procedures of Movat’s pentachrome, safranin O / fast green, H&E and immunohistochemical staining are listed in the Supplementary File (Figures S1 and S2).

### 2.4. Micro-CT and Microscopy

Ex vivo micro-CT images were obtained using vivaCT 80 scanner (Scanco Medical AG, Zurich, Switzerland) at a voltage of 70 kVp and isotropic voxel size of 39 μm, with 114 μA beam current and 350 ms integration time. The radiological data were further processed with Fiji (https://imagej.net/Fiji.html accessed on 14 August 2022) and Voxler (Golden Software, Golden, CO, USA) [15]. The manufacturer’s software for micro-CT allowed density measurement by the application of a bone calibration phantom. Based on the density information, color-mapping could be realized with Voxler by rendering all voxels higher than a certain density value with given color defined in the Colormap Editor. Bright-field microscopic images were taken with a digital slide scanner (MoticEasyScan One, MoticEurope SLU, Barcelona, Spain) at 20× magnification. No further quantitative and statistical analyses were performed for the current preliminary study.

## 3. Results

### 3.1. Staining and Imaging of Rat Femur Fracture Samples Decalcified with EDTA

**Figure 2:**
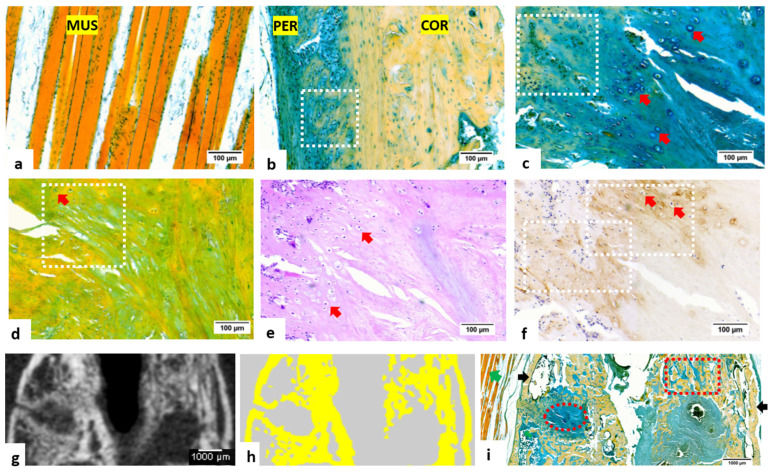
Histochemical and immunohistochemical staining of the femur fracture sample and comparison between micro-CT and multicolor staining images (decalcified with EDTA). (**a**) Multicolor staining of skeletal muscle fibers (MUS), orange to red in color; (**b**) multicolor staining of the cortical bone (COR), showing bone matrix in yellow and adjacent periosteum (PER) blue to green in color; newly formed bone tissue under the periosteum (periosteal reaction) during fracture healing can be observed (white-dashed frame); (**c**) multicolor staining at the fracture site, showing a transitional area with hypertrophic chondrocytes (red arrows), as well as extra-cellular matrix undergoing mineralization (white-dashed frame); (**d**) Movat’s pentachrome staining at the fracture site, with high-mineralized tissue in yellow and low-mineralized tissue in green; the white-dashed frame indicates a transition area undergoing mineralization; (**e**) H&E staining at the fracture site, showing the existence of hypertrophic chondrocytes (red arrows); (**f**) immunohistochemical staining at the fracture site, showing the expression of type-X collagen in extracellular matrix surrounding hypertrophic chondrocytes (red arrows) as evidence of endochondral ossification process; (**g**,**h**) micro-CT of the femur sample at fracture site (slice thickness = 39 μm), with a density-based color-mapping (the area in yellow represented mineralized tissue with a density of higher than 400 mgHA/cm^3^); (**i**) multicolor staining of the fracture site (slice thickness = 10 μm) showing a similar pattern of bone tissue distribution compared with micro-CT imaging. Tissue components including cartilaginous callus, woven bone, cortical bone and adjacent muscle fibers could be identified (black arrows: cortical bone; red-dashed frame: woven bone; red-dashed circle: cartilaginous callus; green arrows: skeletal muscle fibers).

The multicolor staining allowed visualization and identification of the following tissue components in different colors (Figure 2a–c): cortical bone in yellow, skeletal muscle fibers in orange to red and cartilaginous callus in blue to green. In comparison with Movat’s pentachrome staining at the fracture site (Figure 2d), in which mineralized bones were stained in yellow and cartilaginous callus in green, the multicolor staining presented better visualization of cellular details, especially the hypertrophic chondrocytes. Though H&E staining also supported the visualization of cellular details (Figure 2e), the identification of mineralized and non-mineralized tissue components could hardly be realized.

In immunohistochemistry (Figure 2f), the expression of collagen type-X was found in the extracellular matrix surrounding hypertrophic chondrocytes. This region corresponded to the transitional area between cartilaginous callus and mineralized woven bone in multicolor staining (Figure 2c), supporting the application of multicolor staining in studies involving endochondral ossification such as fracture healing [16,17,18].

After obtaining the ex vivo micro-CT images (Figure 2g), a quantitative color-mapping was conducted for presentation of the calcified region with a density of more than 400 mgHA/cm^3^ (Figure 2h). The spatial distribution of yellow-colored high-mineralized tissue components in CT image, namely woven and lamellar bone, corresponded to the yellow-color area on the multicolor staining (Figure 2i), while the histochemical method also allowed visualization of other low- or non-mineralized tissues, which were not effectively rendered in CT images.

### 3.2. Micro-CT Imaging and Histochemical Staining of Tail Vertebra Decalcified with Formic Acid

**Figure 3:**
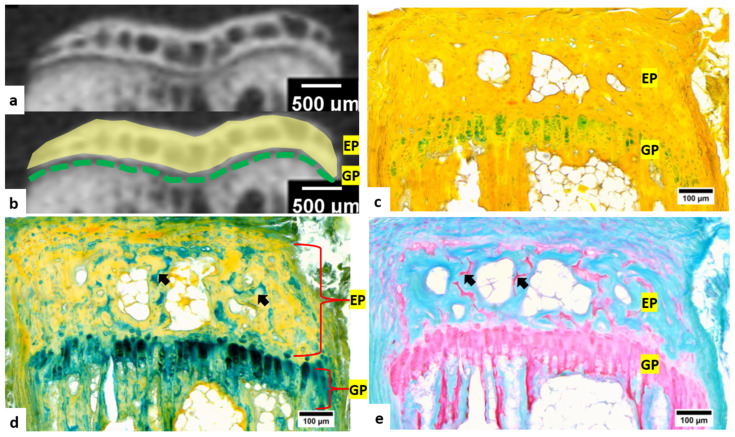
Micro-CT and histochemical staining of the tail vertebra (decalcified with formic acid). (**a**,**b**) Micro-CT images showing radiological regions of growth plate with low-density (GP, green dotted line) and high-mineralized endplate (EP, yellow-labeled area); (**c**) Movat’s pentachrome staining: the endplate and growth plate region could be identified; (**d**) multicolor staining: the bone tissue in endplate were yellow to orange in color, and the cartilaginous growth plates were light-blue to dark-green in color (arrows: cartilaginous components in endplate); (**e**) safranin O / fast green staining, showing a similar pattern of tissue distribution in the bony endplate and cartilaginous growth plate as in the multicolor staining (arrows: cartilaginous components in endplate).

Micro-CT was also conducted for tail vertebra (Figure 3a,b) to identify the bony endplate and cartilaginous growth plate, whose pattern of bone and cartilage distribution were similar to the joints of the limb, thus allowing the evaluation of staining methods for bone and joint samples. In Movat’s pentachrome staining, the bony endplate and cartilaginous growth plate regions could be identified (Figure 3c). However, multicolor staining could not only identify the general contour of endplate and growth plate but also enabled more detailed visualization of the cartilaginous components within the endplate region (arrows in Figure 3d). The cartilaginous tissue could also be distinguished with safranin O and fast green staining (Figure 3e) but was not clearly visible in micro-CT images.

## 4. Discussion

Although there are less invasive imaging methods developed for animal studies of musculoskeletal diseases, the application of these techniques might be limited by various factors, including the accessibility of expensive equipment, difficulty in positioning during imaging especially for large animals, complications resulting from anesthesia, extra burden or stress as a confounding factor for analysis, as well as motion artifacts and ring artifacts that are sometimes unavoidable during in vivo or ex vivo imaging [1]. With relatively easily accessible devices, traditional histological and immunohistochemical staining with optical microscopy are still important in biomedical and clinical research, providing reliable outcomes [6,9,10,19,20].

The multicolor staining method for paraffin-embedded decalcified samples allowed identification of frequently analyzed tissues in animal models of musculoskeletal disorders. The protocol involved relatively few chromogens without requiring long experimental time or usage of hazardous substances; thus, the overall environmental and health risks could be reduced. The polyvalent basic Alcian blue 8GX was selected, aiming at reacting with polysaccharides such as glycosaminoglycans in musculoskeletal samples. The subsequent formation of blue precipitates was widely used for the identification of cartilage and callus tissues in research [21]. Orange G is an acidic synthetic dye with small size and molecular weight, allowing rapid penetration of structures and retaining only in tissues with dense texture such as mineralized bone and muscle fibers. The introduction of phosphotungstic acid in Orange G solution is to maintain the pH level and to intensify the staining effect, which was developed by Papanicolaou for Pap staining [22,23].

Femoral fracture and tail vertebra samples were selected to enroll major types of tissue, including cortical bone, cartilaginous callus, cartilage and skeletal muscle. Additionally, considering the application of different decalcification protocols among laboratories, both EDTA and non-EDTA protocols were tested in this study. The new method provided reliable results in both types of decalcified samples. More staining outcomes are provided in the Supplementary File to further illustrate its performance in samples undergoing different decalcification process (Figure S3) or section thickness (Figure S4). In addition, multicolor staining was also tested effectively in pork rib samples decalcified with formic acid (Figure S5), showing potential application in preclinical research involving large animals. The immunohistochemistry of collagen type-X was analyzed in parallel, showing an efficacy of this method in detecting the transitional area undergoing endochondral ossification during fracture healing [16,17,18]. The multicolor staining in the vertebra samples also effectively detected the existence of cartilaginous tissue in the bony endplate region, comparable to the outcome of safranin O / fast green staining. In addition, micro-CT and density-based color-mapping further validated the staining results for mineralized skeletal tissue. To the authors’ knowledge, this is the first study using a quantitative imaging method for the validation of histochemical staining in decalcified bone samples.

Based on the above results, the authors suggest applying the multicolor staining protocol for skeletal tissue decalcified with formic acid or EDTA, especially for samples including both mineralized and cartilaginous components, or for research involving endochondral ossification. The introduction of manual, semi-automatic or advanced methods based on artificial intelligence for image segmentation and tissue quantification could further enhance the future application of this staining protocol [9,24,25].

## 5. Conclusions

The novel multicolor staining enabled the efficient identification of mineralized and non-mineralized musculoskeletal tissue in paraffin-embedded bone samples decalcified with EDTA and formic acid. The application and future potential optimization of this method may facilitate translational and clinical studies of musculoskeletal disorders.

## Figures and Tables

**Table 1:** List of chemical reagents.

Chemical Reagent	Catalogue Number	Manufacturer	Note
Alcian blue 8GX solution	66011	Sigma-Aldrich, Schneldorf, Germany	histochemical staining
Phosphotungstic acid-Orange G solution	3470	Carl Roth, Karlsruhe, Germany	histochemical staining
Safranin O	84120	Sigma-Aldrich, Schneldorf, Germany	histochemical staining
Fast green FCF	F7258	Sigma-Aldrich, Schneldorf, Germany	histochemical staining
H&E fast staining kit	9194.1	Carl Roth, Karlsruhe, Germany	histochemical staining
Movat’s pentachrome staining kit	12057	MORPHISTO, Frankfurt am Main, Germany	histochemical staining
PBS Tablets	1111.2	Carl Roth, Karlsruhe, Germany	immunohistochemistry
PBS-TWEEN® Tablets	524653-1EA	Merck, Darmstadt, Germany	immunohistochemistry
Triton™ X-100	X100-100ML	Sigma-Aldrich, Schneldorf, Germany	immunohistochemistry
IHC Select Citrate Buffer pH 6.0, 10x	21545	Merck, Darmstadt, Germany	immunohistochemistry
ReadyProbes™ Endogenous HRP and AP Blocking Solution	R37629	Thermo Fisher Scientific, Waltham, MA, USA	immunohistochemistry
Normal goat serum	5425	Cell Signaling Technology, Danvers, MA, USA	immunohistochemistry
Collagen X Monoclonal Antibody (X53)	41-9771-82	Thermo Fisher Scientific, Waltham, MA, USA	immunohistochemistry
Goat anti-Mouse IgG (H+L) Secondary Antibody, HRP	31431	Thermo Fisher Scientific, Waltham, MA, USA	immunohistochemistry
SignalStain® DAB Substrate Kit	8059	Cell Signaling Technology, Danvers, MA, USA	immunohistochemistry
Cytoseal 60	8310-4	Thermo Fisher Scientific, Waltham, MA, USA	coverslipping

## Data Availability

The data that support the findings of this study are available from the corresponding author upon reasonable request.

## Supplementary Materials

The following supporting information can be downloaded at: https://www.mdpi.com/article/10.3390/bioengineering9100488/s1, Figure S1: Staining procedures; Figure S2. Immunohistochemistry protocol for type-X collagen; Figure S3. Multicolor staining of rat tail vertebra samples decalcified with EDTA and formic acid; Figure S4. Multicolor staining of rat distal femur samples with different section thickness; Figure S5. Multicolor staining of pork rib and rat tail vertebra samples decalcified with formic acid.
