# Supplementary material for: Multicolor Histochemical Staining for Identification of Mineralized and Non-Mineralized Musculoskeletal Tissue: Immunohistochemical and Radiological Validation in Decalcified Bone Samples
Source: Bioengineering (Basel). 2022 Sep 21;9(10):488. doi: 10.3390/bioengineering9100488 (PMC9598171; doi:10.3390/bioengineering9100488)
Supplement: Supplementary file 1 [file bioengineering-09-00488-s001.zip › bioengineering-1892179-supplementary.pdf]

Figure S1. Staining procedures

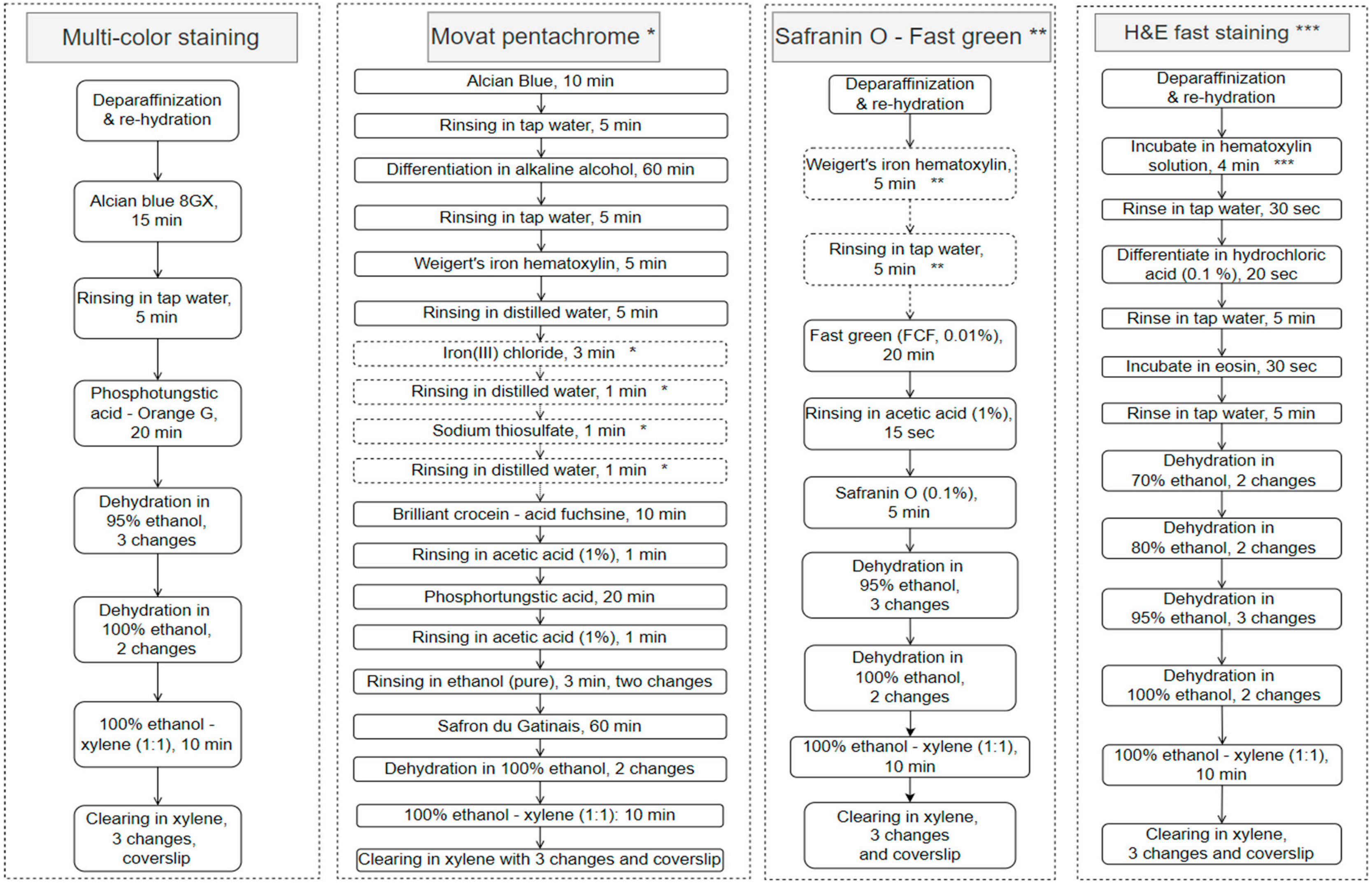

Note:

Deparaffinization and re-hydration: xylene (10 min, 2 changes) → 100% ethanol - xylene (1:1, 20 min) → 100% ethanol (10 min, 2 changes) → 95% ethanol (10 min, 2 changes) → 80% ethanol (10 min, 2 changes) → 70% ethanol (10 min, 2 changes) → rinsing in tap water.

***** with revisions based on procedures in the manufacturer’s manual; procedures labeled with asterisk could be skipped for thin sections of less than 4 μm in thickness.

****** procedures labeled with asterisk could be skipped, according to the research purposes.

******* with revisions based on procedures in the manufacturer’s manual.

Possible optimization for multicolor staining (according to sample status and research purposes): adding Weigert’s iron hematoxylin staining, differentiation in alkaline alcohol, etc.

Figure S2. Immunohistochemistry protocol for type-X collagen

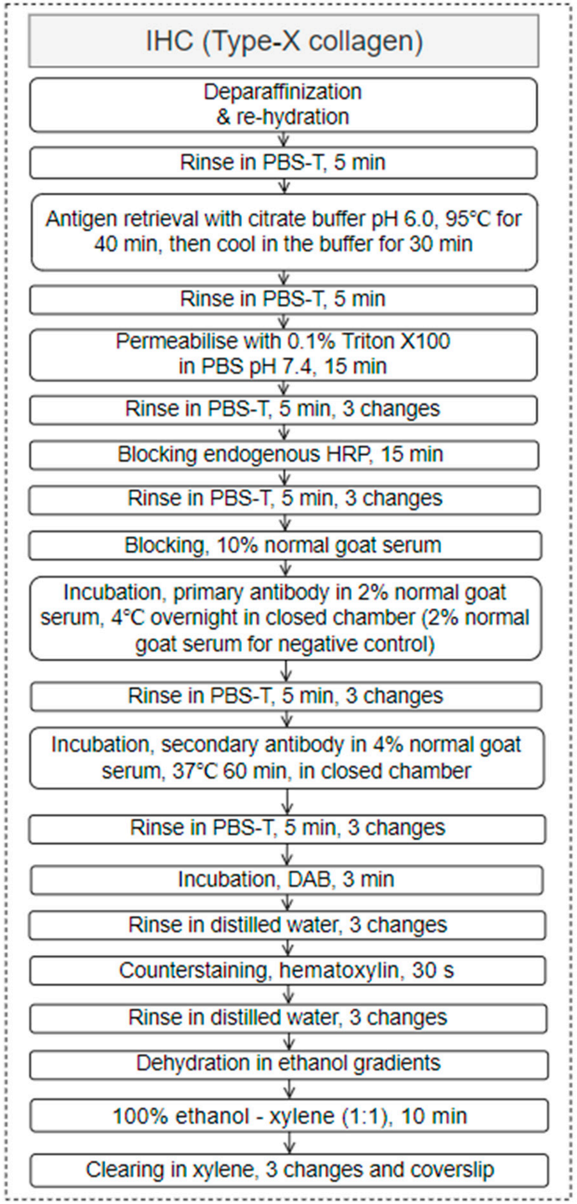

Dilutions for IHC application: primary antibody (1:40), secondary antibody (1:200).

Figure S3. Multicolor staining of rat tail vertebra samples decalcified with EDTA and formic acid.

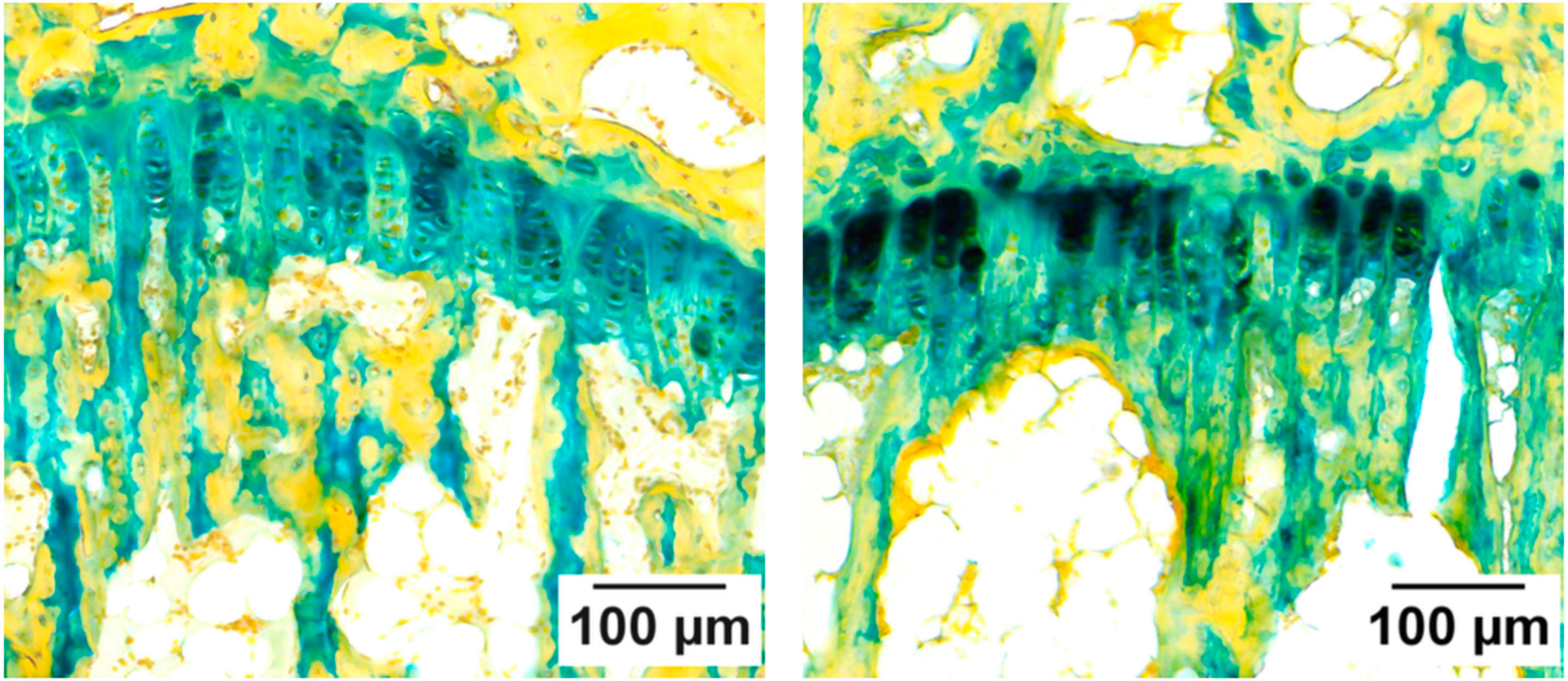

Rat tail vertebra sample, decalcified with 20% EDTA

Rat tail vertebra sample, decalcified with 5% formic acid

Note: section thickness 10 µm.

Figure S4. Multicolor staining of rat distal femur samples with different section thickness.

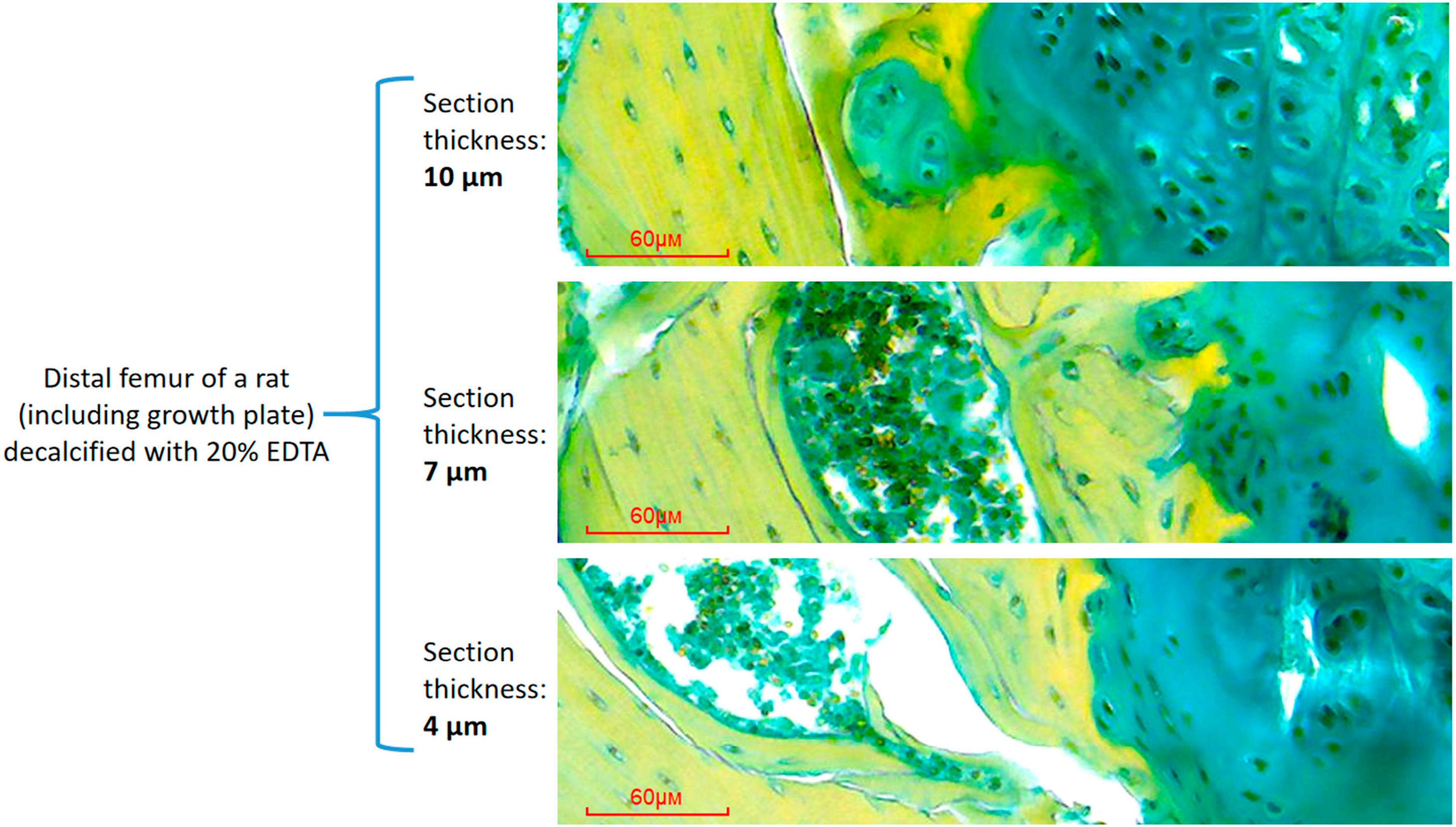

Figure S5. Multicolor staining of pork rib and rat tail vertebra samples decalcified with formic acid.

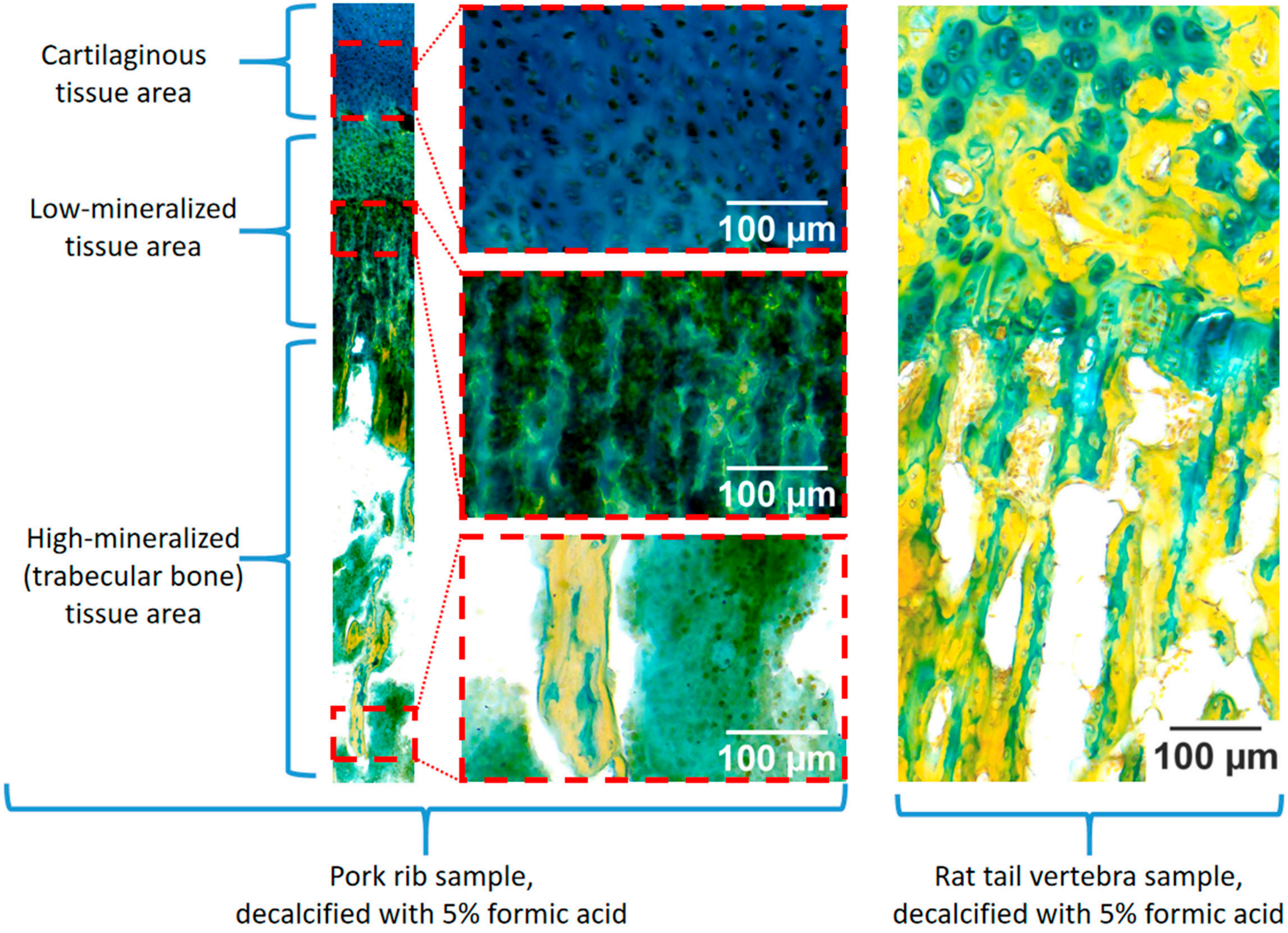

Note: section thickness 10 µm.
